# Executive Function in Adolescence: Associations with Child and Family Risk Factors and Self-Regulation in Early Childhood

**DOI:** 10.3389/fpsyg.2017.00903

**Published:** 2017-06-02

**Authors:** Donna Berthelsen, Nicole Hayes, Sonia L. J. White, Kate E. Williams

**Affiliations:** ^1^School of Early Childhood and Inclusive Education, Queensland University of Technology, BrisbaneQLD, Australia; ^2^Mater Research Institute, University of Queensland, BrisbaneQLD, Australia

**Keywords:** early childhood, parenting, self-regulation, executive function, attention regulation, approaches to learning, adolescence

## Abstract

Executive functions are important higher-order cognitive skills for goal-directed thought and action. These capacities contribute to successful school achievement and lifelong wellbeing. The importance of executive functions to children’s education begins in early childhood and continues throughout development. This study explores contributions of child and family factors in early childhood to the development of executive function in adolescence. Analyses draw on data from the nationally representative study, *Growing up in Australia: The Longitudinal Study of Australian Children*. Participants are 4819 children in the Kindergarten Cohort who were recruited at age 4–5 years. Path analyses were employed to examine contributions of early childhood factors, including family socio-economic position (SEP), parenting behaviors, maternal mental health, and a child behavioral risk index, to the development of executive function in adolescence. The influence of children’s early self-regulatory behaviors (attentional regulation at 4–5 years and approaches to learning at 6–7 years) were also taken into account. A composite score for the outcome measure of executive function was constructed from scores on three Cogstate computerized tasks for assessing cognition and measured visual attention, visual working memory, and spatial problem-solving. Covariates included child gender, age at assessment of executive function, Aboriginal and Torres Strait Islander status, speaking a language other than English at home, and child’s receptive vocabulary skills. There were significant indirect effects involving child and family risk factors measured at 4–5 years on executive function at age 14–15 years, mediated by measures of self-regulatory behavior. Child behavioral risk, family SEP and parenting behaviors (anger, warmth, and consistency) were associated with attentional regulation at 4–5 years which, in turn, was significantly associated with approaches to learning at 6–7 years. Both attentional regulation and approaches to learning were directly associated with executive functioning at 14–15 years. These findings suggest that children’s early self-regulatory capacities are the basis for later development of executive function in adolescence when capabilities for planning and problem-solving are important to achieving educational goals.

## Introduction

Young people who make a successful transition to secondary school, in terms of academic and social adjustment, are also likely to be on track for successful school completion. Currently, there is significant research interest in the contributions of self-regulation and executive function to school achievement for children and adolescents (Blair and Diamond, 2008; Best et al., 2011; Blair and Raver, 2015; Jacob and Parkinson, 2015). The contribution of these abilities to later developmental outcomes is increasingly understood through integration of knowledge across the neurosciences and developmental psychology (Zhou et al., 2012; Diamond, 2013). Executive function, the specific outcome of interest in these analyses, can be defined as higher-order cognitive abilities which are important in goal-directed behavior and which are associated with brain functioning in the prefrontal cortex (Miller and Cohen, 2001; Dumontheil, 2016). Research on the development of executive functions across childhood and adolescence has delivered broad understandings about brain-behavior relationships. This includes knowledge about how different components of executive function mature at different rates and how specialization of brain structure and function in adolescence enables more effective and efficient executive functioning (Davidson et al., 2006). The analyses presented in this paper explore relations between young children’s early family experiences and the self-regulatory behaviors of attentional regulation and approaches to learning, and the development of executive function in mid-adolescence.

Adverse life experiences affect the development of self-regulation and executive function across childhood and adolescence (McEwen and Gianaros, 2010; Sheridan et al., 2012). For example, childhood disadvantage has been found to predict deficits in cognitive processes through the neurological effects of chronic stress (Blair et al., 2011; Evans and Fuller-Rowell, 2013). The experience of chronic stress shapes subsequent stress response physiology in children, leading to higher levels of reactivity and negatively impacting brain development affecting self-regulation and executive function (Evans, 2003). Across early childhood, brain structure and function develop rapidly as children begin to face higher demands for self-regulatory behavior, especially when they make the transition to school (Ursache et al., 2012; Zelazo and Carlson, 2012). Overall, there is increasing knowledge that early life conditions associated with disadvantage affect the development of children’s cognitive processing through childhood and adolescence (Hackman and Farah, 2009; Hackman et al., 2010, 2015).

Early childhood is an optimal period in which early interventions may deliver greater social and individual benefits for long-term development (Heckman, 2006). The early identification of children for whom there are developmental concerns about regulation of behavior, including executive function, is an important research and policy concern across national contexts. For example, since 2009, the Australian Government has conducted a triennial national census of children’s developmental competencies in the first year of school. The Australian Early Development Census (AEDC; Australian Government, 2016) provides national indicators across developmental domains in which self-regulatory behaviors are included. The census identifies the number of children in communities who are ‘vulnerable,’ ‘developmentally at risk,’ or ‘on track’ in language and cognitive skills, communication and general knowledge, physical health and wellbeing, social competence, and emotional maturity. In 2015, it was found that 1 in 5 Australian children were vulnerable in one or more developmental domains and differences in vulnerability were apparent for children with different demographic profiles. This national policy recognizes the importance of readiness to learn when children begin school. It is important that children acquire the necessary skills for cognitive and emotional control in order to become successful learners through the school years (Duncan et al., 2017).

### Self-Regulatory Development during Early Childhood

In these analyses, measures of attentional regulation and approaches to learning that are behaviors associated with self-regulation, are included as possible mediating variables in exploring the longitudinal relations between early childhood disadvantage and family risk factors and adolescent executive function. From a neurological perspective, abilities to control and direct attention that develop across infancy and childhood are the basis of self-regulation (Rothbart et al., 2011; Petersen and Posner, 2012). Increased rapprochement between theories of attentional development and theories of temperament has advanced conceptualizations about the development of self-regulation. Through infancy, there is a transition from attentional reactivity to more voluntary attentional control (Rueda et al., 2004). From 4 to 6 years, increased maturation of the prefrontal cortex provides increased connectivity between neural networks as the basis for attentional regulation. Reactivity and selective attention comprise a dynamic system between the individual’s biological propensities to react and the exercise of attentional control (Ristic and Enns, 2015).

Attentional regulation includes capacities to selectively attend to specific stimuli, inhibit prepotent responses, and monitor actions (Petersen and Posner, 2012). Attentional regulation enables individuals to focus on relevant information to achieve important goals. When children begin school, there are higher demands on attentional regulation and impulse control. These qualities are linked to children’s early academic competence (McClelland et al., 2007; Nesbitt et al., 2013; Blair and Raver, 2015). Williams et al. (2016b) reported that early attentional regulation prior to school, and at school entry, were linked to math achievement at 8–9 years. Longer-term effects of early attention regulation on educational outcomes has been reported by McClelland et al. (2013) who reported that attention span-persistence at aged 4–5 years was predictive of math and reading achievement at age 21 years and college completion at 25 years.

‘Approaches to learning’ has been used as a descriptive term for children’s early self-regulatory skills in the classroom. The construct, approaches to learning (Kagan et al., 1995), has been used in research to describe and measure learning-related, regulatory behaviors that children exhibit when taking part in classroom activities. These behaviors include attention, initiative, persistence, and engagement (Li-Grining et al., 2010; Bulotsky-Shearer et al., 2011; Sasser et al., 2015). If children begin school with behaviors that support engagement, effort, and active participation, successful academic outcomes are much more likely (Fantuzzo et al., 2005; Ziv, 2013).

### Executive Function in Adolescence

The outcome measure in these analyses is executive function which is conceptualized as a single executive control mechanism accounting for high-order thinking. While other areas of the brain are now also implicated in executive functioning, Miller and Cohen (2001) assumed that areas of the prefrontal cortex, associated with executive function, served a particular function to support:

the active maintenance of patterns of activity that represent goals and the means to achieve them. They provide bias signals throughout much of the rest of the brain, affecting not only visual processes but also other sensory modalities, as well as systems responsible for response execution, memory retrieval, and emotional evaluation, etc. The aggregate effect of these bias signals is to guide the flow of neural activity along pathways that establish the proper mappings between inputs, internal states, and outputs needed to perform a given task (p. 171).

Anderson (2003) noted, while executive function may be conceptualized as a single central control mechanism, it is also understood as involving multiple processing systems that are inter-related and inter-dependent. Miyake et al. (2000) investigated the internal factorial structure of executive function across nine tasks to document three distinct but overlapping components of executive function (response inhibition, updating working memory, and set shifting) which has been an influential framework in developmental studies, although in the neurosciences there are broader conceptualizations. In a systematic review of the research literature, Packwood et al. (2011) mapped 68 components of executive function described across 60 studies. Using latent semantic analysis and hierarchical cluster analysis, these researchers identified 18 components that, in turn, represented five sets of complex executive functions involving planning, working memory, set-shifting, inhibition, and fluency.

Adolescence is a period of development that begins at the onset of puberty and spans the second decade of life (Blakemore et al., 2010). While magnetic resonance imaging techniques have found that total brain volume reaches adult levels by puberty (Dumontheil, 2016), brain functions continue to develop and show age-related improvements and differentiation of functions through neural specialization (Luna et al., 2015). Through maturational processes in adolescence, brain processing is seen to become more efficient and effective, despite some recognized vulnerabilities specific to adolescence related to risky behaviors associated with emotional control (Steinberg, 2008). Attentional skills and working memory mature further across adolescence as more complex skills evolve that enable performance monitoring, feedback learning and relational reasoning (Crone and Dahl, 2012). Increased capabilities to integrate more contextual information from experience are also evident in adolescence which permit increased cognitive flexibility for decision-making in accomplishing novel tasks (Steinbeis and Crone, 2016).

### Ecological and Child Factors Influencing the Development of Executive Function

Socio-economic disparities in the measured qualities of executive functions emerge in infancy and across early childhood (Noble et al., 2007; Hackman and Farah, 2009; Blair et al., 2011; Rhoades et al., 2011; Raver et al., 2013) as well as in neurological studies of brain structure and function (Sheridan et al., 2012; Noble et al., 2015). It is less clear if socio-economic disparities in neurological function that have emerged in childhood are maintained over time or if effects are attenuated when children begin school or if family socio-economic circumstances change (Hackman et al., 2015; Duncan et al., 2017).

These analyses consider early family risk factors of maternal mental health, parenting behaviors, and child early behavioral risk as possible influential processes on the development of executive function. A substantial literature has documented links between economic disadvantage and heightened parental depression (Lorant et al., 2003) that, in turn, can impact on parenting and children’s development (Olson et al., 2011). In a review of previous research by Fay-Stammbach et al. (2014), four dimensions of parenting were identified that may impact on the development of executive function: parental home stimulation to support child learning; maternal support and autonomy; parental sensitivity (versus hostility); and control and discipline strategies. Parenting may also be affected by child characteristics, including gender and temperament. Belsky et al. (2007) and Belsky and Pluess (2009) proposed that children differ in their sensitivity to environmental contexts and some children are more reactive to either positive and negative environments which impacts on their behavioral responses. Emerging evidence on such differential susceptibility provides some support that heightened child reactivity can also add stress to the family environment (Raver et al., 2013; Obradovic et al., 2016).

Child behaviors associated with poorer self-regulation at 4–5 years include sleep problems, emotional dysregulation, and inattention/hyperactivity. Early childhood behavioral sleep problems have been linked with poorer attentional regulation (Williams and Sciberras, 2016; Williams et al., 2017) and executive function development over time (Bernier et al., 2013); and also poorer academic functioning (Quach et al., 2009). A recent analysis found that at 4–5 years, children with unresolved behavioral sleep problems, combined with above average levels of emotional dysregulation and poor attention were at higher risk for poor school adjustment (Williams et al., 2016a). Taken together, these findings suggest a link between these early problem behaviors and self-regulation and executive function development over time. Two potential mechanisms or a combination of both mechanisms underpin this link. First, these early problem behaviors may signal an underlying neurological vulnerability for poor self-regulatory functioning. Second, responses by caregivers that fail to resolve early behavioral sleep issues and support positive self-regulation may result in an exacerbation of these problems across childhood. Early sleep problems lead to emotional dysregulation which impacts on attentional regulation, disrupting the development of important brain structures that support executive function (Williams et al., 2017).

### The Current Study

The current study considers the influence of a range of early childhood and family risk factors on the development of executive function in adolescence. While much is known about the impact of family risk on the development of self-regulation and executive function through early childhood, there are fewer studies that have considered how early ecological risk factors and early self-regulatory skills, such as attentional regulation and approaches to learning, may influence the longer-term development of executive function in adolescence.

Path models are developed to explore the direct effects of family socio-economic circumstances, child behavior problems, and maternal parenting behaviors of anger, warmth and consistency, when children are aged 4–5 years, on executive function at 14–15 years. Second, an indirect effects model is developed to examine associations between early ecological risk and executive function in adolescence, through children’s level of attentional regulation at age 4–5 years and their approaches to learning at 6–7 years, when children begin school.

## Materials and Methods

These analyses use data from *Growing Up in Australia: The Longitudinal Study of Australian Children* (LSAC) which commenced in 2004. This cohort study tracks a nationally representative sample of Australian children. It is funded by the Australian Government through a partnership between the Department of Social Services, Australian Institute of Family Studies, and Australian Bureau of Statistics. Ethics approval for the conduct and processes within the study is granted by the Australian Institute of Family Studies Ethics Committee. Detail on LSAC study design, sample information, and implementation is reported in a range of sources (Sanson et al., 2002; Soloff et al., 2005; Gray and Smart, 2009; Edwards, 2012).

The longitudinal Study of Australian Children employs a cross-sequential longitudinal design to follow two cohorts of approximately 5,000 children, aged 0–1 years and 4–5 years. A two-stage clustered sampling design was used to recruit children into the study. Across Australia, 330 postcodes were randomly selected and children for both cohorts were randomly selected from these postcodes. Stratification was used to ensure the number of children in each state/territory and within and outside each capital city was proportionate to the population of children in these areas, except for remote and very remote communities. The sampling frame was derived from the Medicare Australia database held by the Health Insurance Commission which administers this universal health insurance scheme. In 2004 when LSAC commenced, more than 90% of all children born were likely to be registered on the Medicare database by 4 months and 98% by 12 months. Primary data collection occurs through biennial home visits and the study participants include the child, parents (resident and non-resident), and teachers. In these analyses, data are utilized from Wave 1 (2004) when children were 4–5-years-old, Wave 2 (2006) when children were 6–7-years-old, and Wave 6 (2014), when children were 14–15-years-old.

### Sample Selection for Current Study

The current analyses include participants from the 4,983 families initially recruited for the Kindergarten Cohort (4–5 years) in 2004. The current analytic sample was restricted to families for whom the primary parent interviewed at Wave 1 was female and who was a biological or adoptive parent. The resultant sample size was 4819 children and families.

#### Child Characteristics

49.1% (*n* = 2365) of the children are female; mean age at Wave 1 was 57 months (*SD* = 2.64); 3.6% (*n* = 175) had Aboriginal or Torres Strait Islander status; and 12.3% (*n* = 595) spoke a language other than English at home. Compared with the full Kindergarten cohort sample, the selected sample were slightly younger at each wave of data collection than children in excluded families.

#### Maternal Characteristics

2.8% of mothers (*n* = 133) had Aboriginal or Torres Strait Islander status and 15.4% (*n* = 742) had a non-English speaking background. At Wave 1, when children were 4-years-old, mothers ranged in age from 19 to 52 years with a mean age of 34.6 years. There were 41% of mothers who had not completed high school and 44.4% of mothers had completed a tertiary degree, of at least Bachelor level. Compared with the full Kindergarten cohort sample, mothers in the analysis sample were slightly less likely to be Aboriginal or Torres Strait Islander or speak a non-English language at home; and on average had a slightly higher socio-economic position (SEP) at Wave 2 data collection.

### Measures

At Wave 1, when the child was 4–5 years, parental data were from in-home interviews and self-complete questionnaires. Ecological risk measures are: family SEP, child behavior risk index, maternal mental health, and self-report measures for parenting anger, warmth, and consistency. Covariates in the analyses included child sex, age at assessment of executive function, Aboriginal or Torres Strait Islander status, language other than English at home, and a score on a receptive vocabulary measure at age 4–5 years. Additionally, a parent-reported measure for child attentional regulation at age 4–5 years and a teacher-report measure on approaches to learning when children were 6–7 years old were included. From Wave 6, when children were 14–15 years old, data were included from a direct child assessment for executive function using a composite measure derived from three computerized tasks.

#### Socio-Economic Position

Socio-economic position is a derived variable within the LSAC dataset that combines parental report for socio-demographic items for the child’s household: parental occupational prestige, parental education level, and household income (Blakemore et al., 2009). It is weighted according to household composition (e.g., single-parent household; two-parent household). It has an approximate mean of zero and a standard deviation of one. Higher scores indicate higher family SEP.

#### Child Behavior Risk Index

This index was the sum of dichotomized scores on three measures: sleep problems (0 = no; 1 = yes), emotional dysregulation (0 = no; 1 = yes), and inattention/hyperactivity symptoms (0 = no; 1 = yes).

• ***Sleep problems*** were measured with a single parent-report item in which the mother rated whether the child had a sleep problem on a 4-point scale (*no, mild, moderate, or severe problem*). The rating was dichotomized as no/mild = 0 (no sleep problem) versus moderate/severe = 1 (sleep problem).
• ***Emotional dysregulation*** (reverse of emotional regulation) was measured by parent-report on four items from the short form of the Australian Temperament Scales (child version; Prior et al., 1989). Mothers responded to each item (e.g., cries/yells if not bought what they want) on a 6-point scale (1 = *almost never* to 6 = *almost always*). Responses were summed to create a total score. For the current study, internal consistency for the scale was adequate (α = 0.65). The variable was dichotomized into scores < 90th percentile = 0 (no emotional dysregulation) versus scores ≥ 90th percentile = 1 (emotional dysregulation).
• ***Inattention/hyperactivity symptoms*** were assessed on five items from the Hyperactivity-Inattention subscale of the Strengths and Difficulties Questionnaire (Goodman, 2001). Mothers rated items (e.g., restless, overactive, cannot stay still for long) on the typicality of their child’s behavior for the previous 6-month period on a 3-point scale (1 = *not true*, 2 = *somewhat true* and 3 = *certainly true*). The ratings were summed. For the current study, internal consistency for the subscale was moderate (α = 0.74). The variable was dichotomized into scores < 90th percentile = 0 (no hyperactivity problems) versus scores ≥ 90th percentile = 1 (hyperactivity problems).

#### Maternal Mental Health

The Kessler K6 measure, used to assess psychological symptoms, was developed for the United States National Health Interview Survey (Kessler et al., 2002). Mothers rated six items about their current psychological well-being across the previous 4 weeks: nervous; hopeless; restless or fidgety; everything was an effort; so sad that nothing could cheer you up; and worthless. Items were rated on a 5-point scale (1 = *all of the time* to 5 = *none of the time*). An overall score was calculated by summing and averaging the total score resulting in a score ranging from zero to five (α = 0.84). Higher scores indicate poorer mental health.

#### Parenting Anger

Anger was measured using four items adapted from the National Longitudinal Study of Children and Youth (Statistics Canada, 2000). Mothers rated their feelings of anger or frustration toward the child (e.g., How often are you angry when you punish this child?) on a 5-point scale (*never or almost never, rarely, sometimes, often, always or almost always*).

#### Parenting Warmth

Warmth was measured using six items from the Child Rearing Questionnaire (Paterson and Sanson, 1999). Mothers rated their expression of physical affection and enjoyment of the child (e.g., How often do you have warm, close times together with this child?) on a 5-point scale (*never or almost never, rarely, sometimes, often, always or almost always*).

#### Parenting Consistency

Consistency was measured using four items adapted from the National Longitudinal Survey of Children and Youth 1998–1999 (Statistics Canada, 2000). Mothers rated the extent to which they followed through with behavioral consequences for the child (e.g., How often does this child get away with things that you feel should have been punished? - reverse coded). Items are rated on a 5-point scale (1 = *never/almost never* to 6 = *all the time*).

For each of the three parenting constructs, a weighted score was used in the analyses computed from the proportionally adjusted factor score regression weights reported in the LSAC Parenting Measures Technical Report (Zubrick et al., 2014). Higher scores indicate higher maternal anger, warmth, and consistency, respectively.

#### Attentional Regulation (4–5 years)

At Wave 1 data collection, parents completed four items from the persistence subscale of the Short Temperament Scale for Children (Fullard et al., 1984). Items (e.g., When this child starts a project such as a puzzle he/she works on it until it is completed even if it takes a long time) are rated on a 6-point scale (1 = *almost never* to 6 = *almost always*). The scores on this scale were summed to create a total score (α = 0.78) with higher scores indicating stronger attentional regulation skills.

#### Approaches to Learning (6–7 years old)

At Wave 2 data collection, teachers completed six items from a subscale of the Social Skills Rating Scale (SSRS) (Gresham and Elliott, 1990). The response scale ranges from 1 = *never* to 4 = *very often*. The items rate children’s attentiveness, task persistence, eagerness to learn, learning independence, flexibility, and organization. The scale score was the mean of the six items (α = 0.92) with higher scores indicating more positive approaches to learning.

#### Executive Function (14–15 years)

Three computer-based tasks from the Cogstate Assessment Battery (Cogstate, n.d.) were completed by the LSAC study child during the in-home interview at Wave 6 data collection. LSAC interviewers were trained to deliver the tasks from Cogstate protocols. Participants are encouraged to work as quickly as they can and be as accurate as possible.

• The ***Identification task*** is a choice reaction time task that measures visual attention across multiple trials. The subject is required to decide as quickly as possible whether a playing card that is presented face up on the screen is red (YES button) or not (NO button). The cards displayed are either red or black joker playing cards and 30 trials are completed within approximately 2 min. The primary outcome measure is speed of performance, calculated by computing the mean of the log10 transformed reaction time for each correct trial response.
• The ***One Back Memory task*** assesses visual attention and working memory. The cards displayed are red or black playing cards. The subject is required to immediately decide if the card is the same (YES button) as the previous one or not (NO button); NO is always the response in the first trial and 30 trials are presented within approximately 2 min. The primary outcome measure is speed of performance, calculated by computing the mean of the log10 transformed reaction time for each correct trial response.
• The ***Groton Maze task*** is a visuo-spatial, problem-solving task involving feedback monitoring and procedural rule acquisition and application (Pietrzak et al., 2009). Respondents learn a hidden pathway through a 10 × 10 grid of tiles, and move from the top left corner of the grid to the bottom right corner. On the first presentation, the path can be found only by using trial and error. Once the pathway has been uncovered and completed by the participant, the same form of the maze is repeated for four more rounds along the same path. The outcome measure is the total number of errors made in attempting to learn the task across five trials in a single session.

#### Covariates Included in the Analyses

Covariates included in the analyses included child gender (0 = male, 1 = female); child age in months (at 14–15 years data collection; Wave 6); Aboriginal or Torres Strait Islander status (ATSI; 0 = no, 1 = yes); language other than English at home (LOTE; 0 = no, 1 = yes); and a continuous measure of receptive vocabulary assessed when the child was 4–5 years of age, using an adapted version of the Peabody Picture Vocabulary Test (PPVT-III; Dunn and Dunn, 1997) developed for LSAC (Rothman, 2005).

### Data Analysis

#### Executive Function Scoring

Data that did not meet completion or integrity checks on any task were treated as missing data. The Identification and One-Back tasks required participants to complete 75% of test trials to receive a score. On the Groton Maze Task, all five trials were required to be completed. Performance integrity was based on an accuracy score for the Identification and One-Back tasks. Accuracy of performance was computed by taking the arcsine square root of the proportion of correct responses for each task (Integrity failure: Identification task = > 80% of trials; One-Back task = > 70%). For the Groton Maze task, performance integrity failure was defined as >120 errors. An additional filter was also applied to the data for each task in which scores below/above three standard deviations were not included. A composite score for executive function was constructed using the three measures, following procedures described in Maruff et al. (2013). For each task, the mean and standard deviation were computed and standardized. A composite score was computed by averaging the standardized scores for the three tasks; re-standardized using the mean and SD for the composite score; transformed once more so that each had a mean of 100 and a standard deviation of 10, and multiplied by -1 so that higher scores indicated more competent performance. If data on any individual task was missing, the composite score was not computed.

#### Missing Data

The degree of missing data varied by data collection wave as well as by the method used for data collection. Variables collected at Wave 1 using the parent self-complete questionnaire (i.e., measures of emotional dysregulation, inattention/hyperactivity symptoms, maternal mental health, and attentional regulation) had up to 16% of cases with missing data. At Wave 2, the measure on the teacher questionnaire, approaches to learning, had 27% of cases with missing data (38% of these because of participant dropout between Wave 1 and Wave 2; 62% due to teacher non-response). The composite measure for executive function had 45% of cases with missing data (64% of these because of participant drop out between Wave 1 and Wave 6; 36% due to incomplete data). Cases with complete data across all study variables represented a non-random sample of the complete sample for the Kindergarten Cohort: at Wave 1, families had a higher SEP, *F*(1,4801) = 126.31, *p* < 0.001; were less likely to be Aboriginal or Torres Strait Islander, χ^2^(1, N = 4917) = 27.43, *p* < 0.001; or have language other than English at home, χ^2^(1, *N* = 4819) = 68.68, *p* < 0.001; at Wave 6, children were slightly older than the children with incomplete data, *F*(1,3434) = 9.89, *p* < 0.01.

Although missingness was related to the identified socio-demographic variables, it was assumed as missing at random (MAR), that is, not systematically related to the variable value that could have been provided, at least for the substantive variables of interest (Enders, 2010). Multiple imputation in Mplus, Version 7 (Muthén and Muthén, 1998-2012) was employed to create 40 imputed datasets in line with the recommended number for the level of missing data in this study (Graham et al., 2007). The imputation model used all the variables included in the current analyses, as well as a range of auxiliary variables, including additional sociodemographic information (maternal cultural background; SEP at Wave 6 data collection; child age in months across all six waves of data collection); maternal-reported Attentional Regulation at age 6, 8, 10, 12, and 14–15 years; teacher-report data on the measure of Approaches to Learning at age 8, 10, and 12 years; SDQ hyperactivity/inattention symptoms at 6, 8, 10, 12, and 14–15 years; and teacher-ratings of the child’s literacy achievement at age 14–15 years (using scores on the Academic Rating Scale, National Center for Educational Statistics, 2002). All results presented here are pooled results across the 40 imputed datasets, achieved through the TYPE = IMPUTATION analysis available in MPlus Version 7. The analytic models were also run with the non-imputed dataset and there were no substantial differences in findings.

#### Analytic Approach

Path analyses were used to estimate the direct and indirect effects of hypothetically casual relationships among the variables of interest using Mplus Version 7. ***Model 1*** was an unadjusted direct effects model that examined the direct effects of ecological risk variables when children were 4–5 years (i.e., SEP; child behavioral risk index; maternal mental health; maternal parenting – anger, warmth, consistency) on executive function, at age 14–15 years. ***Model 2*** was a fully adjusted direct effects model that included paths from each covariate (child gender; child age in months at 14–15 years; Aboriginal or Torres Strait Islander status; language other than English at home; and child PPVT at 4–5 years of age) to the outcome variable of executive function. For ***Model 3*** all direct and indirect paths were modeled simultaneously. This was a fully adjusted, indirect effects model which included the mediating variables of child attentional regulation (at age 4–5 years) and approaches to learning (at 6–7 years) on relations between early ecological risk and adolescent executive function. In this model, covariates were also assessed in relation to the outcome measure of adolescent executive function (as per Model 2), and each of the mediating variables introduced in Model 3.

Model fit was assessed by three indices: χ^2^ test, RMSEA, CFI. Multiple indices of fit were examined because the chi-square overall goodness-of-fit test statistic is adversely affected by a large sample size (Byrne, 2012). Therefore, a range of other fit indices are usually included to assess model fit (Bentler, 2007). Model fit was also considered using the Comparative Fit Index (CFI) and root mean square error of approximation (RMSEA). For the CFI, a suggested cut-off criteria of values close to or higher than 0.95 have been suggested when using continuous data (Hu and Bentler, 1999). The RMSEA is an absolute fit index which is sensitive to the number of parameters estimated in the model (Steiger, 2009) and the recommended cut-off value for RMSEA is proposed as close to, or lower than 0.06.

## Results

Descriptive statistics, including bivariate correlations between continuous variables used in these analyses are presented in **Table 1**. Correlations were in the expected directions and almost all were significant due to the large sample size. All early childhood ecological risk variables measured at 4–5 years were significantly correlated with executive function, measured at 14–15 years but were small in magnitude. Approaches to learning at 6–7 years was more strongly correlated with executive function (*r* = 0.22; *p* < 0.01) in comparison to the ecological risk variables. Overall, the ecological risk variables had strong significant correlations with attentional regulation ranging in size from *r* = 0.14 (*p* = 0.01) for SEP and maternal warmth to *r* = -0.32 (*p* = 0.01) with child behavior risk.

**Table 1:** Descriptive statistics and correlations for continuous variables in the analyses.

Variable	1	2	3	4	5	6	7	8	9
Mean	0.47	0.00	4.31	2.10	4.50	4.03	3.92	3.22	100.58
*SD*	0.73	1.00	0.63	0.62	0.44	0.80	0.95	0.70	9.98
(1) Child behavior risk									
(2) Socio-economic position	-0.16								
(3) Maternal mental health	-0.24	0.13							
(4) Maternal anger	-0.36	-0.10	-0.31						
(5) Maternal warmth	-0.06	-0.03	0.11	-0.28					
(6) Maternal consistency	-0.26	0.23	0.22	-0.37	0.10				
(7) Attentional regulation (4–5 years)	-0.32	0.14	0.13	-0.23	0.14	0.18			
(8) Approaches to learning (6–7 years)	-0.22	0.19	0.10	-0.16	-0.03	0.13	0.23		
(9) Executive functioning (14–15 years)	-0.13	0.12	0.04	-0.07	-0.02	0.09	0.16	0.22	

*All correlations which are equal to or above 0.03 are statistically significant at *p* < 0.05. Correlations which are equal to or above 0.05 are statistically significant at *p* < 0.01.*

### Path Models

#### Model 1

This model tested the direct relations between early ecological risk variables and executive function in adolescence. There were significant small negative associations between the child behavior risk index and executive function at 14–15 years (*β* = -0.10), indicating a higher behavioral risk score at 4–5 years was associated with poorer executive function at 14–15 years; and a significant but small positive association between SEP and executive function scores at 14–15 years (*β* = 0.09). There were no significant associations between maternal mental health and the three parenting measures (anger, warmth and consistency) and executive function at 14–15 years. The model accounted for 3% of variance in adolescent executive function. This model was ‘just identified’ as the number of data points equaled the number of parameters to be estimated, meaning interpretation of fit indices is not possible because [χ^2^(0) = 0, *p* = 1; CFI = 1; RMSEA = 0].

#### Model 2

The second model tested the direct relations between early ecological risk and executive function, adjusted for child characteristics as covariates in the model. Child gender (*β* = -0.15), home language other than English (*β* = 0.26), and early receptive vocabulary skills (*β* = 0.14) at 4–5 years were all significantly associated with executive functioning at 14–15 years. Aboriginal and Torres Strait Islander status and age at assessment on executive function were not significantly associated with executive function. The associations between the child behavior risk and executive function at 14–15 years (*β* = -0.09), and between SEP at 4–5 years and executive functioning at 14–15 years (*β* = 0.06) remained significant when controlling for child background factors, although effects were slightly attenuated. This model was also ‘just identified’ meaning interpretation of fit indices is not possible, [χ^2^(0) = 0, *p* = 1; CFI = 1; RMSEA = 0].

#### Model 3

**FIGURE 1:**
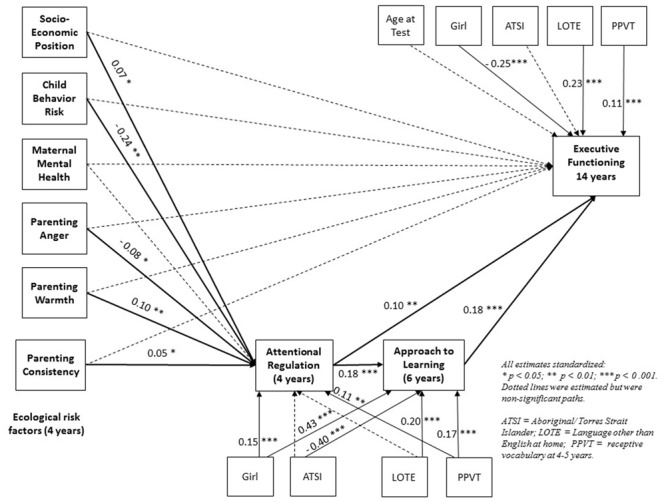
Standardized path model estimates of the relations between early ecological risk, attentional regulation and approaches to learning pathways, and adolescent executive function.

The third model tested the relations between early ecological risk and executive function in adolescence with mediating variables of attentional regulation at 4–5 years and approaches to learning at 6–7 years included, and controlling for child characteristics. The standardized regression coefficients are presented in **Figure 1**. There were statistically significant small associations between child behavioral risk (*β* = -0.24), SEP (*β* = 0.07), maternal anger (*β* = -0.08), maternal warmth (β = 0.10), and maternal consistency (*β* = 0.05) and attentional regulation measured contemporaneously at 4–5 years. The direct associations between child behavioral risk and executive function (*β* = -0.04), and between SEP and executive function (*β* = 0.03) were no longer significant. Maternal mental health was not significantly associated with attentional regulation at 4–5 years or executive functioning at 14–15 years. Attentional regulation at 4–5 years was significantly associated with approaches to learning at 6–7 years (*β* = 0.18). Attentional regulation at 4–5 years (*β* = 0.10) and approaches to learning at 6–7 years (*β* = 0.18) were both directly associated with executive function at 14–15 years.

Overall, the model accounted for 10% of variance in executive function at 14–15 years; 15% of variance in attentional regulation at 4–5 years; and 14% of variance in approaches to learning at 6–7 years; and. The model was an adequate fit to the data [χ^2^(8) = 68.61, *p* < 0.001, RMSEA = 0.04, CFI = 0.95]. The standardized direct, indirect and total effects for each pathway were modeled simultaneously and these effects are presented in **Table 2**. While the total effects for the significant early ecological risk variables are relatively small, the strongest contributions indicated by the total effects on executive function are family SEP, child behavior risk, and attentional regulation.

**Table 2:** Standardized direct, indirect and total effects for full SEM model with executive function as outcome.

	Direct effect	Indirect effect	Total effect
Socio-economic position → Executive function	0.03	0.01^∗∗^	0.04^∗∗^
Behavior risk index → Executive function	-0.04	-0.03	-0.07^∗∗^
Maternal mental health → Executive function	-0.02	0.00	-0.02
Maternal anger→ Executive function	-0.01	-0.01^∗∗^	-0.02
Maternal warmth → Executive function	-0.03	0.01^∗∗^	-0.02
Maternal consistency → Executive function	0.01	0.01^∗∗^	0.02
Attentional regulation → Executive function	0.10^∗∗^	0.03^∗∗^	0.13^∗∗^

*^∗^p < 0.05; ^∗∗^p < 0.01.*

## Discussion

These analyses explored developmental pathways between ecological risk in early childhood and executive function in adolescence. Measures of attentional regulation and approaches to learning were also included in the path models as possible mediating variables between early risk and later executive function skills. In utilizing data from an Australian national study, this research provided opportunity to validate findings from studies conducted in other national contexts about the relations between early risk and the development of executive function across childhood.

In the initial analytic model that examined direct pathways from early childhood to adolescence, higher child behavior risk (i.e., sleep problems, emotional dysregulation, and hyperactivity-inattention problems), lower SEP and child behavior risk were associated with poorer executive functioning in adolescence. This finding aligns with previous studies indicating that early childhood disadvantage and behavior risk impacts on later cognitive control abilities (Evans and Fuller-Rowell, 2013). When the model was adjusted with the covariates related to child characteristics, these direct associations between family socio-economic circumstances and child behavior risk and executive function remained significant. Being male, speaking a language other than English at home, and higher receptive vocabulary scores at age 4–5 years were associated with higher performance on executive function. These specific child characteristics also remained influential on executive function performance in the full, indirect effects model.

When the measures for attentional regulation at 4–5 years and approaches to learning at 6–7 years were also included in the model, attentional regulation had unique and direct effects on adolescent executive function, even when the more proximal variable of approaches to learning measured at 6–7 years was included. Attentional regulation and approaches to learning mediated the relation between early ecological risk and executive function. In relation to the covariates, being female and having higher receptive language competence was associated with higher attentional regulation and being female and speaking a language other than English at home was related to higher scores on approaches to learning. Identifying as Aboriginal or Torres Strait Islander was associated with lower ratings on approaches to learning.

There were no significant direct pathways between maternal mental health and executive function or between the parenting variables and executive function, but indirect paths from these early parenting factors to executive function through attentional regulation and approaches to learning were found in the final model. This indicates that the proximal processes of maternal well-being and parenting practices measured in early childhood had primarily influenced the development of early self-regulatory skills of attentional regulation and approaches to learning at the beginning of school and was a basis for more competent executive function in adolescence.

### Supporting the Early Development of Self-regulatory Skills

The indirect pathways through which ecological factors operated on early self-regulatory skills, and then on executive function are of particular interest. An implication is that interventions aimed at improving adolescent executive function would be best targeted toward improving attentional regulation and approaches to learning in early childhood, rather than waiting until adolescence to intervene. Intervention efforts have focused on improving executive function in adolescence, especially for managing specific cognitive and academic tasks (Jacob and Parkinson, 2015). However, the focus on early self-regulatory skills may yield more and earlier benefits to disadvantaged children, because these skills promote earlier academic success and engagement at the beginning of the school years which is likely to have lasting positive benefits. Further studies that contribute to enhanced understanding about the development of self-regulatory skills in early childhood can provide information about the ‘when’ and ‘how’ of appropriate intervention.

Montroy et al. (2016) reported considerable heterogeneity in the development of self-regulation through ages 3–7 years, using data collated for 1,386 children who participated in three United States studies. For the majority of children, the overall pattern in the development of behavioral self-regulation was a period of rapid development across the preschool year (4–5 years), although the trajectories varied as to when a period of rapid development began and in the rate of growth across the preschool year. This rapid spurt in development during the preschool year was also dependent on the level of behavioral self-regulation that children demonstrated when they entered preschool. Additionally, 20% of the children did not achieve the necessary gains in behavioral self-regulation across the preschool year. Some of these children, at age 6–7 years, were only exhibiting self-regulation skills at the mean level which their peers had achieved at age 4–5 years. In particular, this latter group of children may be children exposed to stressful and adverse family environments and for whom the necessary parenting supports were not available from an early age.

### Child Characteristics: Executive Function, Attentional Regulation and Approaches to Learning

The child characteristics, as covariates included in the modeling, yielded some important associations with executive function and with the mediating variables of attentional regulation and approaches to learning. Child characteristics included in the analyses were gender, Aboriginal and Torres Strait Islander Status, speaking a language other than English at home, and receptive vocabulary scores at age 4–5 years.

With respect to the influence of gender, there is an interesting crossover in the findings. While boys performed more competently than girls on the executive function tasks in adolescence, girls had significantly higher attentional regulation at age 4–5 years, as well as higher teacher ratings for approaches to learning at 6–7 years. These early gender differences with respect to the advantage held by girls during childhood are evident across other studies on the development of self-regulatory skills. Boys appear to lag behind girls in the development of early self-regulation (Kochanska et al., 2001; Matthews et al., 2009, 2014). This suggests that additional supports for boys may be necessary in the early childhood years in order to address gender differences in self-regulatory competence. Suggested explanations for the gender difference have included that boys are more susceptible to adverse environmental conditions than girls and that parents and teachers hold higher expectations for girls for self-regulation than for boys (Montroy et al., 2016). These hypotheses have not been explored extensively in research, including whether gender differences in self-regulation are maintained or diminish beyond the early childhood years.

However, boys significantly outperformed girls on executive function in adolescence. One possible explanation for this finding may be related to the mode of delivery of the executive function tasks as a computer-based assessment and how that mode of assessment might differentiate performance by gender, given boys may have different levels of experience with computer game-playing, as a contextual experience (Desai et al., 2010). Jerrim (2016) conducted cross-national analyses of 2012 data from the *Program for International Assessment* (PISA) for 15 year olds. The analyses involved more than 200,000 adolescents from 32 countries who completed their mathematics assessment through paper-based and computer-based modes of delivery, as a basis for decision-making on changing the mode of delivery. Jerrim (2016) reported that the gender gap varied significantly across the majority of countries, in favor of boys. The average mathematics score for boys was considerably higher than for girls under both assessment modes but the gender gap favoring boys was considerably larger for the computer-based assessment across 20 countries, including Australia. This suggests that the computer-based mode of assessment for adolescent executive function in the current study may account for at least a portion of the gender variance in favor of boys.

Other analytic work with PISA data by Jerrim (2014) also informs interpretation of the current finding with respect to children who spoke a language other than English at age 4–5 years (i.e., indicating a different cultural background to the majority English-speaking Australian population). These children had better performance on executive function as well as higher teacher ratings on approaches to learning, Jerrim investigated why children of East Asian descent in Australia, who were born and raised in Australia and who were second-generation immigrants, outperformed their Australian peers who were not from immigrant families. The East Asian population constitutes the highest proportion of non-English speaking immigrants in Australia. The 2012 PISA data for 15-year-old adolescents for mathematics assessments were examined, as well as a range of other child-report data gathered in PISA assessment including measures of academic motivation, academic effort, time spent studying out of school, work ethic, and a self- control scale. The second-generation East Asian immigrants outperformed their Australian peers in mathematics by more than 100 PISA test points (i.e., equivalent of two and a half years of schooling). Jerrim proposed that a combination of family investments made by parents for their children contributed to this outcome. These factors included family selection of high quality schools, family values placed upon education, family investment in out-of-school tuition, and the adolescents’ high work ethic and high aspirations for their future education, reflecting self-regulatory behaviors.

The LSAC measure for receptive language at 4–5 years was also influential on executive function performance and on parent-reported attentional regulation. Language development is an important child characteristic known to affect the development of self-regulation, although expressive language is most often assessed rather than receptive language, as in the LSAC study. Language competence gives children abilities to organize and categorize information that enable more efficiency in retaining and processing incoming information. However, more research is needed to better understand the relations between the development of language, self-regulation and executive function over time (Bohlmann et al., 2015). Language also is a tool to deal with abstract ideas and propositions in abstract thinking and relational reasoning that is important to executive function in adolescence (Crone and Dahl, 2012; Steinbeis and Crone, 2016).

### Implications for Prevention of Poor Self-Regulation in Early Childhood

In these analyses, the indirect pathways operating from ecological risk through early self-regulation skills to executive function, indicated that children who already exhibited behavioral risk (sleep problems, emotional dysregulation, hyperactivity-impulsivity), whose families had lower socio-economic status, and for whom there may have been maternal mental health issues and poorer parenting, had poorer self-regulation skills (attentional regulation and approaches to learning) in the early childhood years. As Montroy et al. (2016) noted the developmental trajectories for behavioral self-regulation from 3 to 7 years are important. At age 4–5 years and, even before age 4, sufficient family supports can be provided to ensure that children begin school with requisite skills to attend and engage productively in classroom activities. Teachers in early childhood classroom are also in a position to first recognize children’s inabilities to focus attention, follow instructions, and persist in completing tasks when they begin school. These self-regulatory behaviors are malleable and can be addressed with the right supports for children, their families, and teachers.

The Australian Government initiative to identify the incidence and prevalence of vulnerable children in the first year of school using data from the AEDC (Australian Government, 2016) is an important first step but more understanding is needed on how to use this data to target the most vulnerable children for intervention and family support programs who have problems with language, cognitive, and communication skills, and who lack social competence, and emotional maturity. For example, Goldfeld et al. (2017) in an analysis of AEDC identified that mental health competence is unequally distributed across the Australian child population at school entry and is strongly predicted by measures and correlates of disadvantage. It is important to intervene early with children who demonstrate early behavior risk at 4 years, including sleep problems, emotional dysregulation (high reactivity) and hyperactive-impulsive behaviors as measured in the current study as part of child behavioral risk. Other research (Williams and Sciberras, 2016; Williams et al., 2017) indicates the reciprocal relations among these behaviors from an early age. Sleep problems across the early childhood period, in particular, may drive and exacerbate emotional and attentional dysregulation. Interventions that address early sleep problems could be explored in order to reduce children’s behavior risk when beginning school and may have downstream benefits for executive function development.

### Strengths and Limitations

A strength of this research lies in the use of longitudinal data from a large national study. The analyses also used different sources of data that included parent report, teacher report, and direct child assessment. However, the national representative sample does not represent a low income or disadvantaged population in line with more specific US studies that have used highly selected samples from disadvantaged populations or samples with wide income diversity (Bradley et al., 2001). The relatively advantaged population in the current study may explain the smaller estimates and effect sizes in the associations between socio-economic status and adolescent outcomes for executive function. The causal relationships between socioeconomic status and executive function have not yet been fully explored and this may only be possible with well-designed intervention studies.

Furthermore, it is acknowledged that the parent-report measure of attentional regulation when the child was 4–5 years had a degree of conceptual and measurement commonality with an item used in computation of the Child Behavior Risk Index. This item was based on parent-report on the SDQ subscale scores for inattention/hyperactivity symptoms, for which a clinically significant cut-point (≥90%) was used to create a binary item indicating high risk. This was summed with other binary risk items similarly constructed for sleep problems and emotional dysregulation. In comparison, the attentional regulation measure comprised a summary score for four items rated on a 6-point scale that focused on persistence and employed positively framed items about attentional behaviors (e.g., When this child starts a project such as a puzzle he/she works on it until it is completed even if it takes a long time).

Additional limitations of the study include a lack of fine-grained measurement of self-regulatory behaviors in childhood which would usually include measurement of inhibitory control (Rhoades et al., 2009) and working memory (Simmering, 2012). Furthermore, the components of the model of executive function used in this study were somewhat different from components assessed in many other child development studies that have a strong focus on inhibitory control, including using effortful control as a primary theoretical model (Zhou et al., 2012). The measures of executive function available in this secondary dataset had less focus on emotional control involved in solving complex and novel tasks.

While the benefits of secondary data analysis with large longitudinal datasets include access to large samples with multiple time points of data collection, these advantages are often offset by the possible breadth and depth of measurement. Future studies could include more breadth of measurement of self-regulation and executive function, at more frequent time points, across childhood and adolescence. Such studies will be able to explicate the nature of developmental pathways involving ecological risk, self-regulatory behaviors and executive function in adolescence.

## Conclusion

Executive function is a set of neurocognitive processes that allow individuals to achieve short- and long-term goals, particularly when they are required to adjust their thinking and their actions as environmental demands change (Crone and Dahl, 2012). The development of executive function and associated self-regulatory skills across childhood and adolescence are important to later successful adjustment and achievement (Moffitt et al., 2011; Diamond, 2013). In these analyses, while the effects of the early ecological risk on the development of executive function were relatively small, they operated through children’s early self-regulatory behaviors of attentional regulation and approaches to learning, at the beginning of the school years. The research findings have identified possible directions for early intervention to enhance self-regulatory competence in early childhood in order to ensure later capabilities for executive control in adolescence.

## Author Contributions

DB, NH, SW, and KW contributed to the initial development of the theoretical models. NH, SW, and KW contributed to different parts of the data preparation and data analysis. DB drafted the manuscript, with input from NH, SW, and KW. All authors approved the final version of the manuscript.

## Conflict of Interest Statement

The authors declare that the research was conducted in the absence of any commercial or financial relationships that could be construed as a potential conflict of interest.
